# Titanium nitride coating of pectus bar increases metal contamination after minimally-invasive repair of pectus excavatum

**DOI:** 10.1371/journal.pone.0292616

**Published:** 2023-10-12

**Authors:** Caroline Fortmann, Thomas Göen, Soeren Wiesner, Jan Hegermann, Rim Kiblawi, Martha Dohna, Benno M. Ure, Diane Miriam Renz, Claus Petersen, Joachim F. Kuebler

**Affiliations:** 1 Department of Pediatric Surgery, Hannover Medical School, Hannover, Germany; 2 Social and Environmental Medicine, Institute and Outpatient Clinic of Occupational, Friedrich-Alexander-Universität Erlangen-Nürnberg, Erlangen, Germany; 3 Hannover Medical School, Institute for Biostatistics, Hannover, Germany; 4 Hannover Medical School, Research Core Unit Electron Microscopy, Institute of Functional and Applied Anatomy, Hannover, Germany; 5 Department of Pediatric Radiology, Hannover Medical School, Institute of Diagnostic and Interventional Radiology, Hannover, Germany; Hamadan University of Medical Sciences, ISLAMIC REPUBLIC OF IRAN

## Abstract

**Introduction:**

Previous studies demonstrated a release of toxic metals, e.g. nickel and chromium, from stainless steel bars used for minimally invasive repair of pectus excavatum (MIRPE). In the present study, we investigated the impact of titanium nitride coating on the metal release and exposure of MIRPE patients.

**Material and methods:**

We analyzed the courses of nickel and chromium levels in blood, urine and local tissue in patients undergoing MIRPE with a titanium nitride coated pectus bar between 03/2017 and 10/2018. Sample collection was scheduled prior to MIRPE, at defined postoperative time points and at bar removal. Additionally, we evaluated irritative symptoms. Results were compared to a control group who received uncoated stainless steel bars in a previous time period (03/2015–02/2017).

**Results:**

12 patients received coated pectus bars (mean age 15.7 years). The control group included 28 patients. After implantation of a titanium nitride coated bar, significant increase in systemic nickel and chromium levels after one, two and three years was noted. In an interim analysis one year after MIRPE, we observed patients with coated bars to have significantly elevated trace metal values compared to the control group. This elevation persisted throughout the observation period. Tissue metal values were also significantly increased. Irritative symptoms occurred significantly more often in study patients compared to controls (50.0% vs. 14.3%).

**Conclusions:**

Coating of pectus bars with titanium nitride failed to reduce metal contamination after MIRPE. Instead, it resulted in a significant increase of trace metal levels after MIRPE, compared to patients with stainless steel bars, which may be explained by wear of the coating and inter-component mobilization processes.

## Introduction

Allergic reactions following minimally-invasive repair of pectus excavatum (MIRPE) have been frequently reported in recent years [1–6]. As a potential cause for these irritative symptoms a contamination with nickel and chromium has been demonstrated [7–10]. Localized soft-tissue reactions and dermatitis are known to be caused by these metals [11, 12]. These findings called for a technical solution in order to reduce the metal contamination and thereby provoked side-effects.

One option to lower the release of metals is material substitution. In orthopedics, chromium and cobalt contamination after metal-on-metal (MoM) hip arthroplasty has been successfully reduced by using metal-on-polyethylene implants instead [13, 14]. Pectus bars consist of stainless steel and could be substituted by titanium. This material is already used for MIRPE in patients with known metal allergy. However, titanium bars must be pre-bent by the manufacturer to match the individual shape, intraoperative re-shaping is difficult and the costs are four- to five-fold higher [2–5].

Another common method to render implants in orthopedics hypoallergenic is coating of a standard implant [15]. The often-used titanium-nitride (TiN) coating is biocompatible and has a high surface resistance to abrasion and corrosion [16, 17]. The use of TiN surfaces has been shown to reduce the release of the underlying material [18].

In an attempt to reduce nickel and chromium contamination in our patients, we began using newly developed TiN coated bars for pectus repair and analyzed local and systemic metal levels after implantation. Additionally, we documented irritative symptoms related to the bar in these patients. The aim of the study was to analyze the amount of metal contamination when using TiN coated bars and their clinical tolerance.

## Patients and methods

As a response to our data demonstrating high nickel and chromium contamination in patients after MIRPE [19], MedXpert (Eschbach, Germany) developed TiN coated pectus bars and stabilizers. We changed our MIRPE protocol from stainless steel bars to these coated bars from the same manufacturer. The patients receiving these new bars in our department of pediatric surgery of the Hannover Medical School from March 2017 until October 2018 were prospectively included into our analysis. The metal levels were compared to the data from our previously published patient cohort with stainless steel bars [19]. The institutional review board approved this study (No. 6695), and all patients and legal guardians gave their written informed consent. During the clinical period, data were not made anonymous, but statistical analysis was performed with anonymous data.

### Patients’ characteristics, operative technique, and follow-up

Patients’ characteristics were prospectively collected and included age, sex, size and number of bars, and history of metal allergy. In case of a suspected metal allergy, a coated allergy test plate provided by the manufacturer was applied. MIRPE was performed as previously described [10]. Implantation of one or more coated bars was performed under thoracoscopic guidance. During individual shaping with a pectus bar tabletop bender we protected the coated bar with a soft plastic bag to prevent damage of the coating. Bars were fixed on the lateral chest wall using coated stabilizers. However, slight movements were possible between the stabilizers and the bar due to the fact that the stabilizers were not fixed to the bars, just a pin at the end of the bars prevented dislocation of the stabilizers. Follow-up was scheduled four weeks, as well as one and two years following MIRPE. Approximately three years after pectus surgery, the bars were removed in general.

### Evaluation of irritative symptoms

Irritative symptoms were evaluated at every follow-up time point and included rash, pleural effusion, seroma, local swelling, wound healing disorder, persistent pain, and lassitude, defined as previously described [19]. Independent of the scheduled follow-up, patients with irritative symptoms were seen as required in our outpatient clinic.

### Bar composition

The producer (MedXpert, Eschbach, Germany) used titanium nitride for coating of the stainless steel bars and stabilizers. Coating was performed using physical vapor deposition, and a layer of 1.3–3.0 μm was superimposed at 180–250° Celsius. Vickers Hardness Number was up to 2400 and coefficient of friction was 0.5. Coating of the bars was carried out in a validated process for compliance with the parameters established for the execution. This ensured a uniformly implemented coating that met the quality requirements. Testing and confirmation was carried out via test samples per production lot. The titanium coated bars were cleared for medical use from the medical device certification center but only available in our department for the evaluation period.

### Method of sample collection for metal analysis

According to our previous data on nickel and chromium contamination after MIRPE using a stainless steel bar [19], we analyzed nickel and chromium levels in blood, urine and tissue at the standardized time points. Utilized collection tubes and cannulas were free of any metal contamination. Local subcutaneous tissue samples were collected intraoperatively before implantation. During bar explantation, local tissue samples were extracted directly adjacent to the stabilizer. Blood and urine samples were taken before surgery and at the follow-up time points. In patients who had to travel long distances, the family physician collected blood and urine samples in the postoperative period.

### Metal analysis

Analysis of chromium and nickel was performed at the Institute of Occupational, Social and Environmental Medicine of the Friedrich-Alexander-Universität Erlangen-Nürnberg, as previously described [10, 19]. Briefly, the chromium and nickel content present in patients’ tissue, urine, and blood was assessed using an inductively coupled plasma mass spectrometer (ICP-MS) with collision cell (Agilent 7500cx). Monitoring of the ion mass 52 (chromium) and 60 (nickel) was used for quantification. The accuracy of the chemical analyses was ensured by the successful participation in the German External Quality Assessment Scheme [20].

### Appraisal of metal levels

Reference values for metal levels in blood and urine have been published by the *Deutsche Forschungsgesellschaft* (*DFG) Commission for the Investigation of Health Hazards of Chemical Compounds in the Work Area* and other sources indicating the general metal level in the general German population (nickel in blood 0.5 μg/l, nickel in urine 3 μg/l, chromium in plasma 0.4 μg/l, chromium in urine 0.6 μg/l) [21]. When the individual levels were above the reference values, this was defined as trace metal contamination. In combination with irritative symptoms, it was considered as intoxication.

### Scanning electron microscopy

We used scanning electron microscopy on unbent and bent coated bars to evaluate microscopic defects on the coating surface. 1 cm pieces of the bars were sawed out and glued onto scanning electron microscope (SEM) stubs. Imaging was performed in a Zeiss Crossbeam 540 (Zeiss, Oberkochen, Germany) at 10 kV and a working distance of 7 mm. Samples were scanned parallel to the longitudinal axis of the bars.

### Imaging

In one patient who developed wound healing disorder three months after removal of his two pectus bars, we performed magnetic resonance imaging (MRI) of the chest in a Siemens Avanto Magnetom 1.5T MRI system (Siemens Healthineers, Erlangen, Germany). The following sequences were acquired: transverse diffusion weighted images; transverse (fat saturated) and coronal (without fat saturation) T2-weighted motion insensitive, multi-shot turbo spin echo sequences (BLADE); T1-weighted gradient echo 3D volume interpolated breath-hold examination (VIBE) sequences, before (transverse in DIXON technique) and after intravenous application of contrast agent (fat saturated in coronal and transverse orientation).

### Data analysis

Statistical analysis was performed using SigmaStat^®^ by Jandel Scientific. Data were reported as median and interquartile range for continuous variables and as percentages for categorical variables. Based on the comparison to the mean values of the control group we additionally reported the mean and standard error of the mean for continuous variables. Statistical analyses were performed using the one-way analysis of variance on ranks and Dunn’s method for posthoc analysis of each follow-up time point to baseline. P<0.05 was defined to indicate statistical significance. Additionally, trace metal levels were compared to a control group of patients who underwent implantation of an uncoated stainless steel bar for MIRPE, using the Dunn’s method. Comparison of the incidence of irritative symptoms in each group was performed using Fisher exact test.

## Results

We included 12 patients who underwent MIRPE with a coated bar between March 2017 and October 2018. Mean age of the patients at the time of MIRPE was 15.7 years (range 14–18 years), 91.7% were male. All patients denied personal or family history of metal allergy preoperatively. Seven patients (58%) received one pectus bar, five patients (42%) received two bars which were implanted in parallel orientation. The mean length of the bars was 11 inches (±0.3; range 9–13 inches). Bar removal was scheduled after a mean of 36 months (±1.0; range 31–42 months) and performed from November 2019 until January 2022. In one patient the bar remained in place during the observation period.

The control group included 28 patients (mean age 16.4 years, 92.2% male) who underwent MIRPE using stainless steel bars without coating at our department between March 2015 and February 2017 [19]. The protocol for sample collection and metal analysis was identical in patient and control group. After initial enrollment of 37 patients in the control group, eight patients had to be excluded due to incomplete data at bar removal and one patient was excluded due to early bar removal. 60.7% of the analyzed 28 patients received one bar, 35.7% two bars and 3.6% three bars. Pectus bars were explanted in the control group after a mean time of 36 months (± 0.75; range 30 to 52 months).

### Systemic metal values

**Fig 1:**
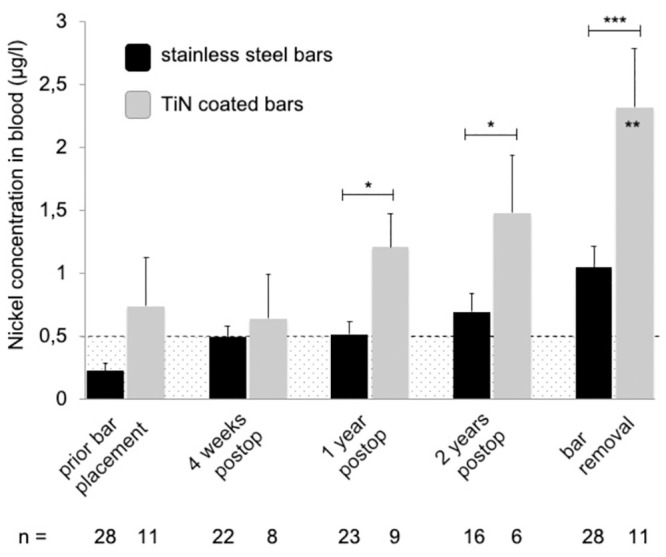
Mean nickel levels in blood following MIRPE. Mean nickel concentrations in blood (stainless steel group in black, TiN coated bar group in grey), error bars show standard error of the mean. Hatched area indicates values below the reference values. The postoperative concentrations in the TiN coated bar group were compared to preoperative values; the overall difference was statistically significant (*p = 0*.*0239*). Additionally, values at each time point were compared to the corresponding time point of stainless steel bars (*p<0.05; **p<0.01; ***p<0.001). Postop = postoperative, TiN = titanium-nitride, MIRPE = minimally-invasive repair of pectus excavatum.

**Fig 2:**
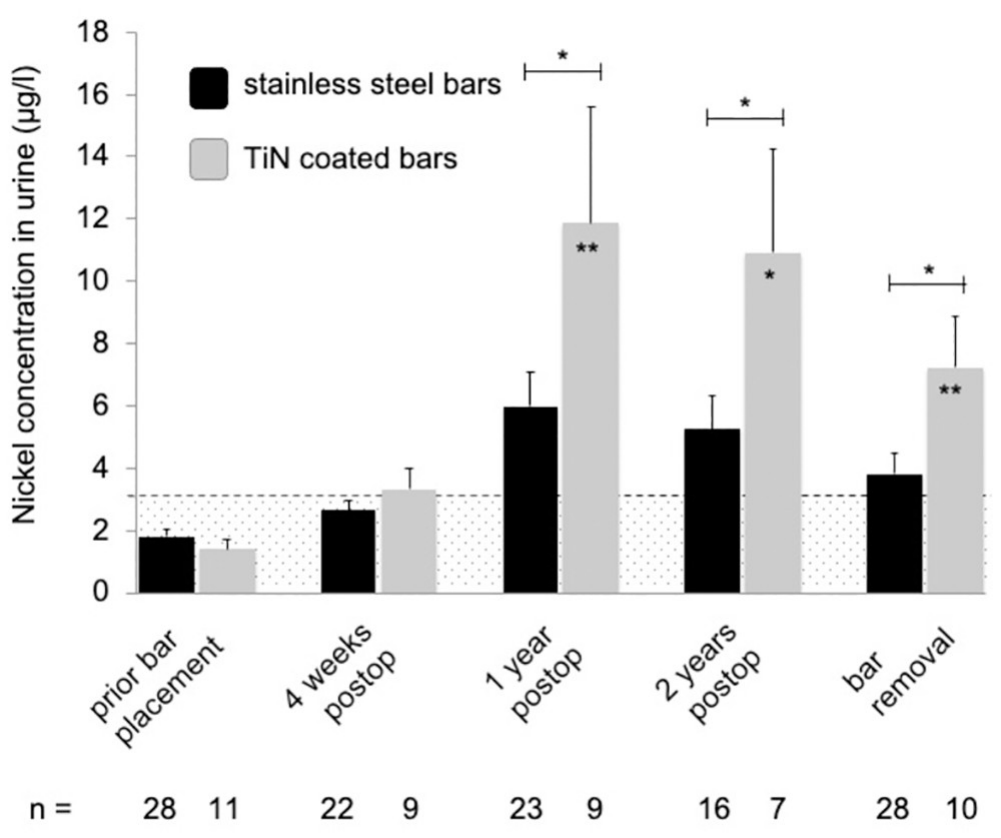
Mean nickel levels in urine following MIRPE. Mean nickel concentrations in urine (stainless steel group in black, TiN coated bar group in grey), error bars show standard error of the mean. Hatched area indicates values below the reference values. The postoperative concentrations in the TiN coated bar group were compared to preoperative values; the overall difference was statistically significant (*p = 0*.*0038*). Additionally, values at each time point were compared to the corresponding time point of stainless steel bars (*p<0.05; **p<0.01). Postop = postoperative, TiN = titanium-nitride, MIRPE = minimally-invasive repair of pectus excavatum.

**Fig 3:**
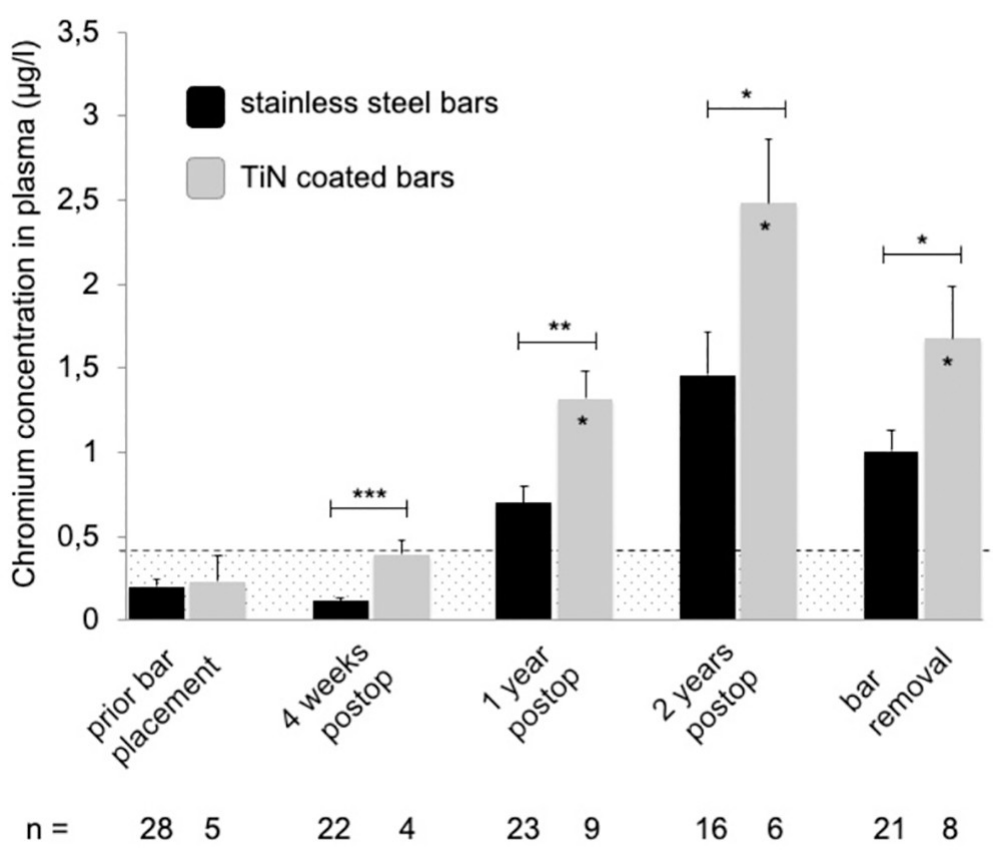
Mean chromium levels in plasma following MIRPE. Mean chromium concentrations in plasma (stainless steel group in black, TiN coated bar group in grey), error bars show standard error of the mean. Hatched area indicates values below the reference values. The postoperative concentrations in the TiN coated bar group were compared to preoperative values; the overall difference was statistically significant (*p<0*.*001*). Additionally, values at each time point were compared to the corresponding time point of stainless steel bars (*p<0.05; **p<0.01; ***p<0.001). Postop = postoperative, TiN = titanium-nitride, MIRPE = minimally-invasive repair of pectus excavatum.

**Fig 4:**
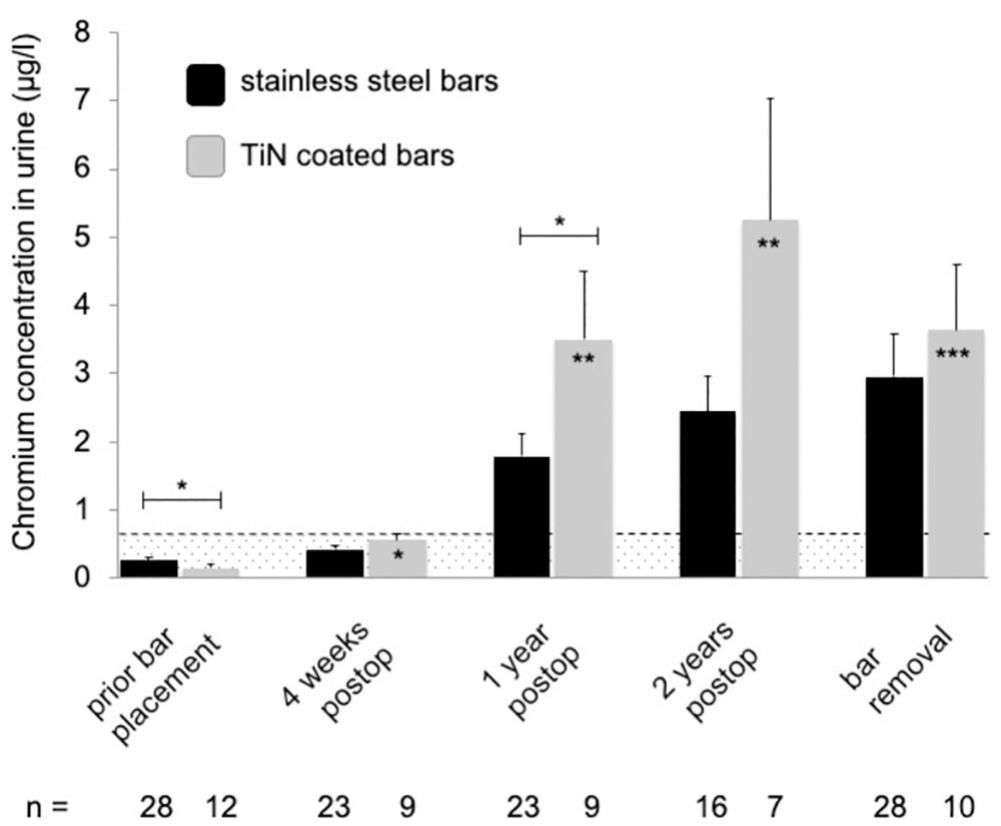
Mean chromium levels in urine following MIRPE. Mean chromium concentrations in urine (stainless steel group in black, TiN coated bar group in grey), error bars show standard error of the mean. Hatched area indicates values below the reference values. The postoperative concentrations in the TiN coated bar group were compared to preoperative values; the overall difference was statistically significant (*p<0*.*001*). Additionally, values at each time point were compared to the corresponding time point of stainless steel bars (*p<0.05; **p<0.01; ***p<0.001). Postop = postoperative, TiN = titanium-nitride, MIRPE = minimally-invasive repair of pectus excavatum.

Almost all mean systemic metal levels were significantly elevated and exceeded the reference values at follow-up time points one, two and three years after MIRPE, compared to preoperative mean values (*p<0*.*05*) (Table 1 and Figs 1–4). Additionally, no patient had normal metal levels. Median metal levels are also displayed in Table 1. One year post MIRPE, we compared the systemic mean metal levels in our patient group with coated bars to our control group with stainless steel bars and found a significant increase in the coated bar group (*p<0*.*05*) (Figs 1–4). Upon this data we discontinued the use of coated bars in our department for ethical reasons. The significant difference between both groups lasted until bar removal.

**Table 1:** Mean and median metal levels at standard time points.

	preop	4 weeks postop	1 year postop	2 years postop	bar removal
**Nickel in blood** (μg/l)	0.74* (±0.39) ---------- 0.5 [0.00–0.62]	0.64* (±0.35) ---------- 0.24 [0.00–0.79]	1.21* (±0.26) ---------- 1.26* [0.68–1.65]	1.48* (±0.46) ---------- 1.01* [0.98–1.33]	2.32* (±0.47) ---------- 1.83* [1.45–2.09]
**Nickel in urine** (μg/l)	1.39 (±0.31) ---------- 0.91 [0.68–2.00]	3.34* (±0.63) ---------- 3.37* [2.10–4.54]	11.87* (±3.72) ---------- 8.07* [4.78–10.60]	10.91* (±3.34) ---------- 7.26* [4.13–18.07]	7.24* (±1.61) ---------- 5.16* [3.44–10.89]
**Chromium in plasma** (μg/l)	0.23 (±0.15) ---------- 0.00 [0.00–0.47]	0.39 (±0.09) ---------- 0.40 [0.29–0.50]	1.32* (±0.16) ---------- 1.40* [0.90–1.70]	2.48* (±0.38) ---------- 2.48* [2.11–2.58]	1.88* (±0.34) ---------- 1.60* [1.10–2.60]
**Chromium in urine** (μg/l)	0.15 (±0.05) ---------- 0.15 [0.00–0.25]	0.56 (±0.10) ---------- 0.51 [0.41–0.63]	3.50* (±1.00) ---------- 2.57* [1.55–3.26]	5.25* (±1.79) ---------- 4.60* [1.81–6.90]	3.64* (±0.97) ---------- 2.46* [1.65–4.98]
**Nickel in tissue** (μg/g)	0.7 (±0.20) ---------- 0.65 [0.61–0.91]				5168.40 (±700.10) ---------- 5709.28 [4353–6198]
**Chromium in tissue** (μg/g)	1.56 (±0.62) ---------- 1.28 [1.12–1.47]				6410.78 (±750.24) ---------- 6473.21 [5780–7354]

Mean metal levels (± standard error of the mean) are shown in the upper part of each table element. Median metal levels and [interquartile range] are mentioned below. *Value above the reference value. Postop = postoperative

### Local metal values

**Fig 5:**
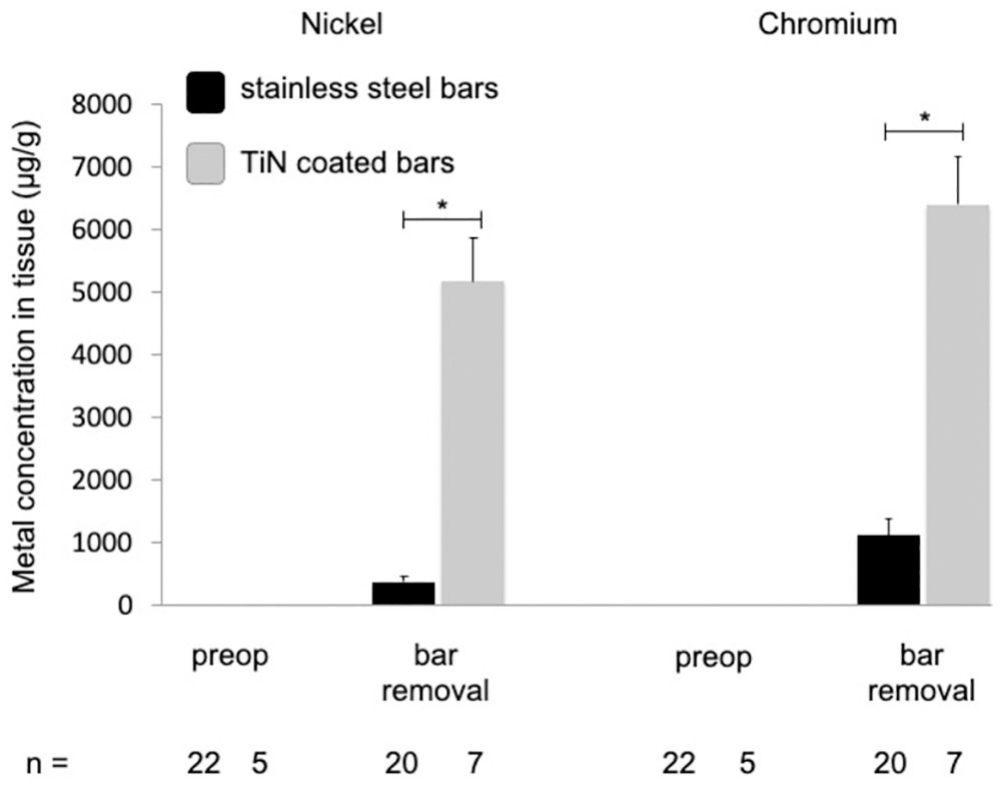
Nickel and chromium levels in local tissue before MIRPE and at time of bar removal. Mean concentrations of nickel and chromium in tissue (stainless steel group in black, TiN coated bar group in grey), error bars show standard error of the mean. Values at bar removal were compared to stainless steel bars (*p<0.05). Postop = postoperative, TiN = titanium-nitride, MIRPE = minimally-invasive repair of pectus excavatum.

**Fig 6:**
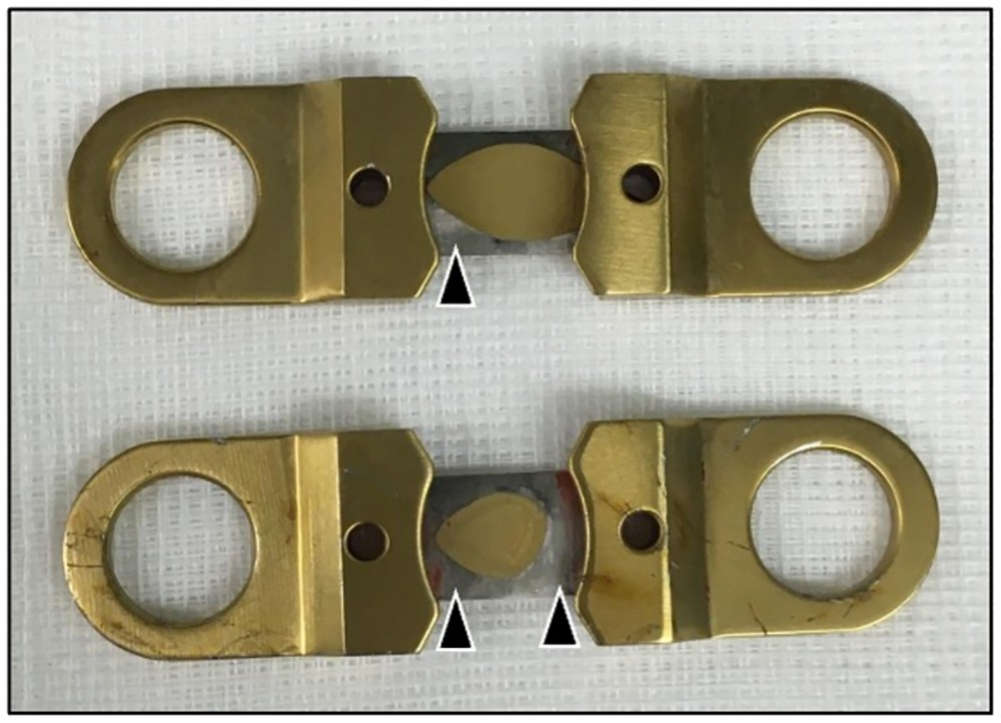
Explanted TiN coated stabilizers. Parts of the golden TiN coating are rubbed off at the region of contact surface with the bar, uncovering the underlying stainless steel (arrowheads). TiN = titanium-nitride.

Nickel and chromium tissue levels also showed a high increase at bar removal, compared to values prior to MIRPE (Table 1). Unfortunately, statistical analysis of these data was not possible as the number of paired data at both time points was too small due to incomplete sample collection. Nevertheless, when comparing local metal levels at bar removal between both groups, we detected a highly significant increase in the group with coated bars (Fig 5). We frequently found a major local tissue reaction around the stabilizers with discolored tissue and bony overgrowth. The stabilizers themselves presented with signs of wear debris of the coating due to continuous small movements on the bar. Thereby, parts of the underlying stainless steel became visible (Fig 6).

### Follow-up

Due to non-compliance of some patients, follow-up data were incomplete (75% after four weeks and after one year, 58% after two years).

### Appearance of irritative symptoms

Six patients (50%) developed irritative symptoms in the early postoperative period, which consisted in persistent pain in five patients, rash in one patient, seroma in one patient, wound dehiscence in one patient, and lassitude in two patients. Symptoms occurred after a mean time of 5.5 weeks after MIRPE (±1.5, range 2–12 weeks). Treatment involved antibiotics in five patients (1.8g clindamycine daily in four patients, 4.5g ampicillin/sulbactam daily in one patient) and additionally 50mg prednisolone daily in three patients. Compared to the control group, in which 13.5% of patients developed irritative symptoms, the incidence of these symptoms was significantly higher in our study group with coated bars (*p = 0*.*041*).

### Electron microscopy

**Fig 7:**
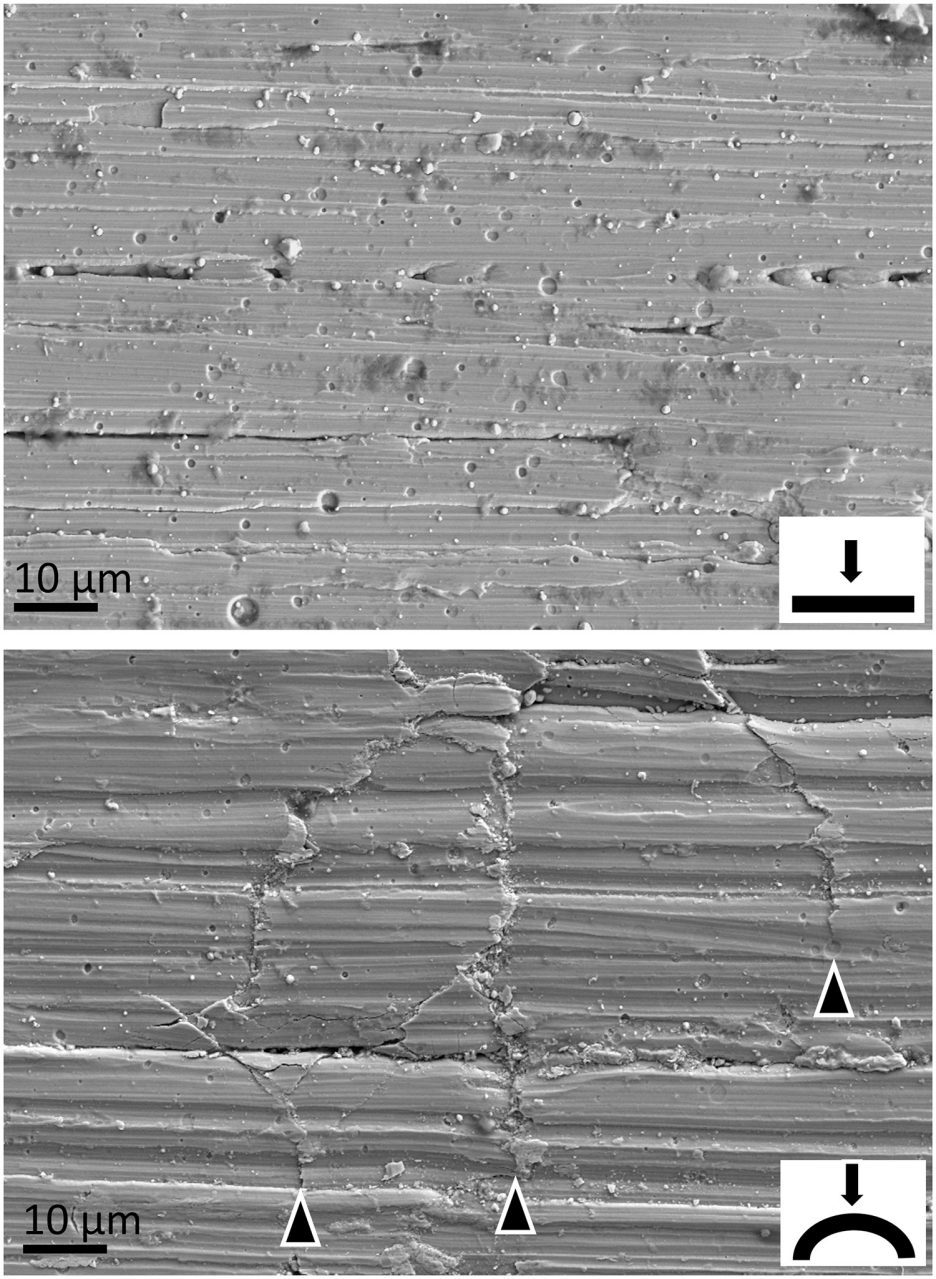
Scanning electron microscopy of TiN coated pectus bars. Top: unbent, below: bent longitudinally. The longitudinal axis of each bar is oriented horizontally. The point of view is depicted in the insets. Note the cracks in the surface of the bent bar (arrowheads), which are oriented mostly crosswise to the bending direction and are absent in the unbent bar. TiN = titanium-nitride.

Using electron microscopy, we detected microscopic defects on the TiN coating surface after bar bending (Fig 7), whereas unbent coated bars did not show similar defects. Microscopic cracks were visible all over the surface in the region of bar bending. The cracks were mostly oriented crosswise to the longitudinal axis of the bar. Plastic wrapping protection of the bent coated bars did not result in less defects on the microscopic level after bending.

### Imaging

**Fig 8:**
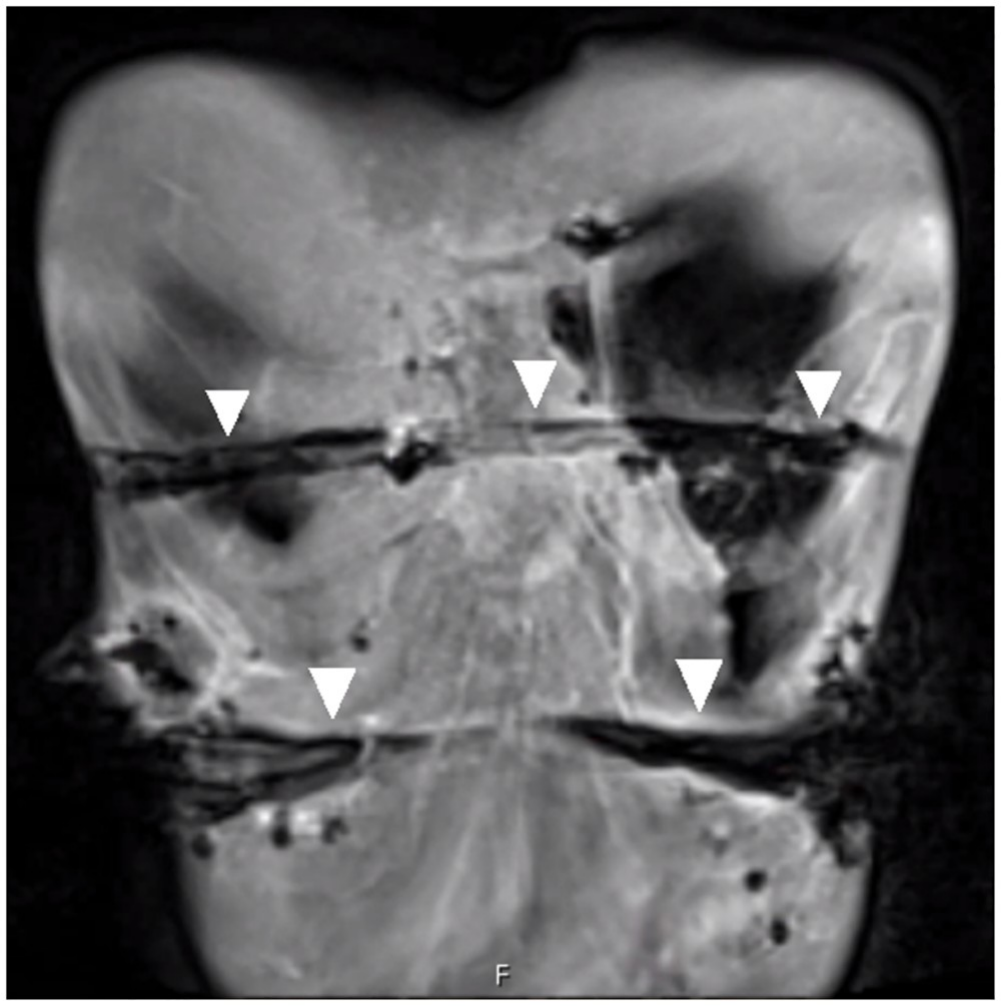
Thoracic MRI three months after removal of two TiN coated pectus bars. Coronal T1-weighted fat saturated 3D volume interpolated breath-hold magnetic resonance examination sequence after intravenous application of contrast agent shows hypointense metal susceptibility artifacts along the former bar tunnels (arrowheads). TiN = titanium-nitride.

One patient developed wound healing disorder of the right lower thoracic wall three months after bar removal and underwent MRI imaging to evaluate for abscess. In both T1- and T2-weighted sequences, hypointense metal artifacts along the former bar tunnels could be observed, creating a pseudoimage in the shape of the two former bars. These findings were interpreted as trace metal wear along the remaining fibrotic bar tunnels (Fig 8). However, artifacts were minor and only impacted image quality restricted to the former bar tunnels without image distortion and with imaging quality of adjacent soft tissue structures sufficient for evaluation [22, 23]. An abscess was not detected.

## Discussion

Metal ions put pectus excavatum patients undergoing MIRPE at risk, as these otherwise mostly healthy patients are exposed to metals related to the implanted bar [7–10, 19]. MIRPE is a standard procedure all over the world, and every year numerous pectus excavatum patients undergo implantation of stainless steel bars. Nickel and chromium ions have been detected in local tissue, blood and urine of patients. This metal contamination appears to be responsible for the irritative reactions increasingly reported after MIRPE [19]. Consequently, a reduction of this contamination is necessary.

Solutions for decrease of metal release have already been developed in orthopedic surgery. After MoM joint replacement, patients were exposed to significantly elevated chromium and cobalt levels caused by wear of the prostheses [24, 25]. This wear was observed especially on the edges of the prostheses [26]. Wear and metal sensitivity were found as a potential causes of implant failure [27, 28]. This clinical problem as well as the metal contamination could be reduced by change of the material to metal-on-polyethylene [13, 14]. Another effective protection against wear debris following orthopedic procedures can be coating of the regular implant with TiN [17, 28]. Thomas et al. demonstrated TiN coating to reduce the metal ion release from the metal substrate potentially preventing cutaneous and peri-implant allergic reactions [29]. Blood chromium levels in patients after coated total knee arthroplasty were significantly lower compared to patients receiving an uncoated prosthesis [15]. In addition, coated prostheses did not show inferior postoperative clinical outcomes in comparison to uncoated implants [15, 17, 30].

Inspired by these data, we started using TiN coated bars for MIRPE. However, in the postoperative period we found significantly elevated nickel and chromium levels in blood and urine samples of patients. These values were even significantly higher than in our control group after implantation of stainless steel bars. On some of the explanted TiN coated stabilizers we found macroscopic edge wear as already reported after orthopedic procedures.

The observed increased release of metals might be explained by wear of the coating, uncovering and ultimately grating of the underlying stainless steel. One reason for this wear could be frequent breathing movements resulting in continuous slight movements of the stabilizers on the bar. Rubbed-off TiN coating particles could be trapped between the surfaces and serve as a third body, resulting in further damage of the surfaces. Third body wear due to defects in the coating layer has been described before [16]. Another possible reason for this wear might be the bending act of the bars during MIRPE. Bending caused microfractures of the TiN surface as we detected using scanning electron microscopy. Protection of the bars with a soft plastic bag during bending maneuvers did not prevent these microfractures in the deeper layers. Microscopic defects of the TiN coating after knee replacement have been reported by other authors [31]. Microfractures of the TiN coating along the entire bar as well as mechanical shear and wear during therapy interval of three years, but also mechanical stress at bar removal might have created slight but sufficient trace metal wear along the bar pathway to generate hypointense artifacts as observed in MRI after bar removal. A reason for the elevated release of metal ions from the coated stainless steel bars compared to the uncoated bars may be the difference of ionization potential of different metals resulting in a faster release of metal ions when in contact to another.

Other options to minimize metallic wear have been reported in the literature. In a joint simulator, use of chromium nitride coating could reduce metallic wear [32]. Advanced superlattice ceramic coating resulted in decreased metal ion levels, compared to uncoated MoM surfaces [33].

In our study cohort, postoperative irritative symptoms occurred in more patients compared to our control group. As we found significantly higher metal levels and a higher incidence of irritative symptoms in the patient group one might hypothesize that higher metal levels lead to or even induce irritative symptoms.

Limitations of our study are the small sample size due to early termination of the study as we discontinued the implantation of coated bars. Additionally, the follow-up between MIRPE and bar removal is not complete. Furthermore, the individual intake of nickel and chromium was not measured and could not be quantified. Preoperatively, mean nickel level in blood was above the reference value in our study group without apparent explanation.

## Conclusion

Titanium nitride coating of pectus bars and stabilizers failed to reduce metal contamination after MIRPE. Instead, nickel and chromium levels in blood and urine of patients were significantly increased, compared to the already elevated values of patients having received regular stainless steel bars without coating. Irritative symptoms have also been observed more frequently in patients with TiN coated bars. The pectus bar industry is in demand for developing an effective solution to reduce metal release of pectus bars.

## Supporting information

S1 File (XLSX)
